# Effect of maternal vitamin D_3_ supplementation on maternal health, birth outcomes, and infant growth among HIV-infected Tanzanian pregnant women: study protocol for a randomized controlled trial

**DOI:** 10.1186/s13063-017-2157-3

**Published:** 2017-09-04

**Authors:** Christopher R. Sudfeld, Karim P. Manji, Christopher P. Duggan, Said Aboud, Alfa Muhihi, David M. Sando, Fadhlun M. Alwy Al-beity, Molin Wang, Wafaie W. Fawzi

**Affiliations:** 1 Department of Global Health and Population, Harvard T.H. Chan School of Public Health, 665 Huntington Ave, Building I Room 1103C, Boston, MA 02115 USA; 2 Department of Epidemiology, Harvard T.H. Chan School of Public Health, Boston, MA USA; 3 Department of Nutrition, Harvard T.H. Chan School of Public Health, Boston, MA USA; 4 Department of Biostatistics, Harvard T.H. Chan School of Public Health, Boston, MA USA; 5 Department of Pediatrics, Muhimbili University of Health and Allied Sciences, Dar es Salaam, Tanzania; 6 Department of Microbiology and Immunology, Muhimbili University of Health and Allied Sciences, Dar es Salaam, Tanzania; 7 Department of Obstetrics and Gynecology, Muhimbili University of Health and Allied Sciences, Dar es Salaam, Tanzania; 8 Division of Gastroenterology, Hepatology, and Nutrition, Boston Children’s Hospital and Harvard Medical School, Boston, MA USA; 9 Management and Development for Health, Dar es Salaam, Tanzania

## Abstract

**Background:**

Vitamin D has significant immunomodulatory effects on both adaptive and innate immune responses. Observational studies indicate that adults infected with HIV with low vitamin D status may be at increased risk of mortality, pulmonary tuberculosis, and HIV disease progression. Growing observational evidence also suggests that low vitamin D status in pregnancy may increase the risk of adverse birth and infant health outcomes. As a result, antiretroviral therapy (ART) adjunct vitamin D_3_ supplementation may improve the health of HIV-infected pregnant women and their children.

**Methods/design:**

The Trial of Vitamins-5 (ToV5) is an individually randomized, double-blind, placebo-controlled trial of maternal vitamin D_3_ (cholecalciferol) supplementation conducted among 2300 HIV-infected pregnant women receiving triple-drug ART under Option B+ in Dar es Salaam, Tanzania. HIV-infected pregnant women of 12–27 weeks gestation are randomized to either: 1) 3000 IU vitamin D_3_ taken daily from randomization in pregnancy until trial discharge at 12 months postpartum; or 2) a matching placebo regimen. Maternal participants are followed-up at monthly clinic visits during pregnancy, at delivery, and then with their children at monthly postpartum clinic visits. The primary efficacy outcomes of the trial are: 1) maternal HIV disease progression or death; 2) risk of small-for-gestational age (SGA) births; and 3) risk of infant stunting at 1 year of age. The primary safety outcome of the trial is incident maternal hypercalcemia. Secondary outcomes include a range of clinical and biological maternal and child health outcomes.

**Discussion:**

The ToV5 will provide causal evidence on the effect of vitamin D_3_ supplementation on HIV progression and death, SGA births, and infant stunting at 1 year of age. The results of the trial are likely generalizable to HIV-infected pregnant women and their children in similar resource-limited settings utilizing the Option B+ approach.

**Trial registration:**

ClinicalTrials.gov identifier: NCT02305927. Registered on 29 October 2014.

**Electronic supplementary material:**

The online version of this article (doi:10.1186/s13063-017-2157-3) contains supplementary material, which is available to authorized users.

## Background

Antiretroviral therapy (ART) is the principal tool utilized for prevention of mother-to-child transmission (PMTCT) of human immunodeficiency virus (HIV) worldwide, which is primarily effective due to its ability to reduce maternal viral load during antenatal, delivery, and lactation periods [1]. In 2015 the World Health Organization (WHO) updated their PMTCT guidelines to universally recommend Option B+ in which all HIV-infected pregnant women initiate lifelong triple-drug ART, irrespective of clinical or immunologic status [2]. In addition to avoiding a CD4 T-cell based ART initiation criterion, additional rationales for utilizing Option B+ include that pregnant women take triple antiretrovirals for their own health, ART prevents mother-to-child transmission of HIV for the current, as well as future, pregnancies, and mothers also avoid the risks associated with starting and stopping antiretrovirals [3]. Due to evidence from the Malawian experience with Option B+ [4], the HIV care and treatment program in Dar es Salaam, Tanzania, started utilizing the Option B+ approach in 2013.

The public health approach of Option B+, if universally implemented, may virtually eliminate mother-to-child HIV transmission, but will also lead to an estimated 1.5 million children per year being born HIV-exposed uninfected (HEU) and exposed to triple antiretrovirals in utero [5]. In resource-limited settings, HEU children have an estimated two- to three-times risk of mortality as compared with children born to HIV-uninfected women [6, 7]. HEU children also appear to be at increased risk of poor birth outcomes and postnatal growth faltering as compared with HIV-unexposed children [8, 9]. A few potential mechanisms, independent of maternal sociodemographic differences, which may lead to increased risk of poor health outcomes among HEU children include exposure to antiretroviral drugs, increased exposure to pathogens, and deficits in immune responses to vaccination and pathogens, [8, 10, 11]. There is also growing evidence that exposure to triple antiretrovirals in utero among HEU children may lead to increased risk of postnatal growth faltering [8, 12]. A recent study in Botswana found HEU infants exposed to triple antiretrovirals in utero had lower length at birth, and at 6 and 24 months of age compared to HEU infants exposed to zidovudine monotherapy [12]. As a result, low-cost ART adjunct interventions are needed to improve birth outcomes and linear growth of HEU children in resource-limited settings.

Vitamin D is a potent immunomodulator which plays a prominent role in the control of intracellular pathogens [13, 14]. Therefore, HIV-infected individuals with low levels of vitamin D may have poorer control of HIV replication and immunologic response to other intracellular infections, notably *Mycobacterium tuberculosis* [15]. Multiple observational cohort studies in both high-income and resource-limited settings have determined that HIV-infected adults with low vitamin D status are at increased risk of mortality, incident tuberculosis (TB), and HIV progression [16–19]. To the best of our knowledge, members of our research group have conducted the only cohort study of vitamin D and clinical outcomes among HIV-infected pregnant women [17]. In this study of HIV-infected pregnant Tanzanian women conducted prior to the introduction of ART, women with a serum 25-hydroxyvitamin D (25(OH)D) concentration < 32 ng/mL in the second trimester (12–27 weeks gestation) had 25% increased risk of progression to WHO HIV stage III disease, and these morbidity benefits appeared to increase linearly up to 70 ng/mL [17]. Women in the highest quintile of 25(OH)D also had 42% lower risk of all-cause mortality as compared to those in the lowest quintile. As a result, provision of ART adjunct vitamin D supplementation may improve the health of HIV-infected pregnant women, even in equatorial settings where levels of deficiency may be low.

In addition to the maternal benefits, children born to HIV-infected mothers with adequate levels of vitamin D in pregnancy and during lactation may also experience health benefits. The only published study of maternal vitamin D in pregnancy and child health outcomes among HIV-infected pregnant women is the previously mentioned cohort study of Tanzanian women who did not receive ART conducted by members of our group [17, 20]. We found that children born to mothers with low vitamin D concentrations in the second trimester (25(OH)D < 32 ng/mL) had significantly increased risk of stunting, being underweight, and reporting cough during their first 5 years of life [21]. There was also some indication that low vitamin D levels were associated with increased risk of small-for-gestational age (SGA) infants, but results were not statistically significant [20].

In contrast to the sparse data among HIV-infected pregnant women, a relatively large number of observational maternal vitamin D studies have been conducted among HIV-uninfected women, primarily in high-income settings. A meta-analysis of 24 observational studies conducted among HIV-uninfected women determined that 25(OH)D levels < 20 ng/mL were associated with an approximate 50% increased risk of SGA and preterm delivery [22]. Despite supportive observational evidence, few randomized controlled trials (RCTs) of vitamin D supplementation have been published to date [23–27]. A trial conducted by Brooke and colleagues among 126 pregnant women of Asian descent in the UK found some indication that 1000 IU vitamin D/day may decrease the risk of SGA, but results were not statistically significant [23]. Similar nonsignificant benefits on SGA were also found in another small trial conducted among 180 pregnant women in the UK which utilized a single bolus dose of 200,000 IU vitamin D or a daily vitamin D_3_ supplement of 800 IU vitamin D as compared to placebo [26]. In a recent randomized trial conducted among 160 Bangladeshi pregnant women, infants born to women supplemented with 35,000 IU vitamin D_3_ per week in the third trimester of pregnancy had significantly increased length-for-age *z*-scores (LAZ) at 1 year of age compared to infants born to mothers receiving placebo [25]. A small trial of maternal vitamin D supplementation in Iran also determined that pregnant women with 25(OH)D < 30 ng/mL at 24–26 weeks gestation randomized to 50,000 IU vitamin D_3_ per week or 400 IU vitamin D_3_ per day had significantly increased birthweight compared to those receiving placebo [24]. A recent meta-analysis of RCTs found that vitamin D supplementation in pregnancy significantly increased birth weight and birth length but had no effect on preeclampsia, gestational diabetes, SGA, preterm birth, and cesarean section [27]. Overall, small randomized trials suggest that vitamin D supplementation may significantly improve some birth outcomes; however, these trials were likely underpowered to detect moderate differences in maternal and child health outcomes and their small sample size also limits the generalizability of their findings.

The WHO currently does not recommend routine vitamin D supplementation in pregnancy owing to a lack of sufficient evidence for an effect on maternal and child health outcomes [28]. In order to address this evidence gap, we present the protocol for an ongoing randomized, double-blind, placebo-controlled trial of maternal vitamin D_3_ supplementation conducted among HIV-infected pregnant women in Dar es Salaam, Tanzania.

## Methods/design

**Fig. 1:**
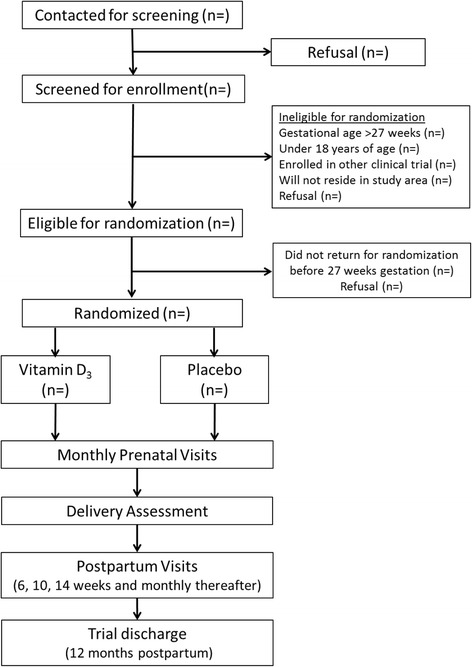
The Trial of Vitamins-5 (ToV5) flow diagram

**Fig. 2:**
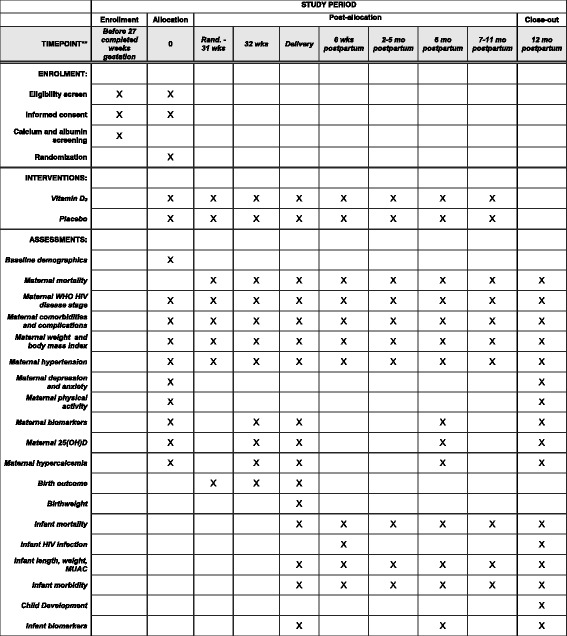
Schedule of enrollment, interventions, and assessments (SPIRIT Figure). *25*(*OH*)*D* 25-hydroxyvitamin D, *HIV* human immunodeficiency virus, *MUAC* mid-upper arm circumference, *WHO* World Health Organization

The Trial of Vitamins-5 (ToV5) is an individually randomized, parallel group, double-blind, placebo-controlled trial of vitamin D_3_ (cholecalciferol) supplementation conducted among 2300 HIV-infected pregnant women receiving ART in Dar es Salaam, Tanzania (ClinicalTrials.gov identifier NCT02305927). The trial protocol was developed by collaborators at the Harvard T.H. Chan School of Public Health in the United States and Management and Development for Health (MDH) and Muhimbili University of Health and Allied Sciences (MUHAS) in the United Republic of Tanzania. The ToV5 protocol diagram is presented in Fig. 1. The schedule of trial enrollment, interventions, and assessments is presented in Fig. 2. The first participant was enrolled in the trial in June 2015 and follow-up data collection will continue through June 2019. This protocol was written following the Standard Protocol Items: Recommendations for Interventional trials (SPIRIT) checklist (see Additional file 1).

### Primary and secondary objectives

The goal of ToV5 is to investigate vitamin D_3_ as a simple and low-cost ART adjunct intervention to improve health outcomes for HIV-infected pregnant women and their children. The primary aims of the trial are to determine the effect of maternal vitamin D_3_ supplementation starting in the second trimester and continuing until 12 months postpartum on: 1) maternal HIV disease progression or death; 2) risk of small-for-gestational age births (below tenth percentile birthweight for gestational age by sex using the Oken standard [29]); and 3) risk of infant stunting (LAZ < –2) at 12 months of age as compared to a matching placebo regimen. The secondary aims of the study are to determine the effect of the maternal vitamin D_3_ supplementation regimen on: 1) maternal CD4 T-cell reconstitution and HIV viral load; 2) maternal immunologic biomarkers (interleukin (IL)-2, IL-12, interferon (IFN)-γ, and the antimicrobial peptide cathelicidin); 3) maternal weight gain in pregnancy; 4) maternal depression and anxiety; 5) maternal hypercalcemia (serum albumin adjusted calcium > 2.6 mmol/L); 6) miscarriage; 7) stillbirth; 8) preterm birth; 9) birthweight; 10) low birthweight (<2500 g); 11) mother-to-child transmission of HIV; 12) infant mortality; 13) infant growth trajectory the first year of life (LAZ, weight-for-age *z*-scores (WAZ), and weight-for-length *z*-scores (WLZ); 14) infant wasting (WLZ < –2); 15) infant underweight (WAZ < –2); 16) infant morbidities and hospitalization; 17) infant cognitive, motor, and socioemotional development; 18) serum 25(OH)D levels in the mother and in the infant during the first 12 months of life; and 19) maternal and infant parathyroid hormone (PTH) concentrations.

### Study population

Women are eligible for ToV5 if they meet all the following inclusion criteria: 1) aged ≥ 18 years; 2) received an HIV-positive test; 3) receiving ART; 4) pregnant and of 12–27 weeks gestation (second trimester) as determined by the report of the last menstrual period (LMP); and 5) albumin-adjusted calcium levels in the normal physiologic range (<2.6 mmol/L). Women were excluded if they: 1) did not intend to stay in Dar es Salaam for 2 years after enrollment; 2) were enrolled in any other clinical trial; or 3) did not provide informed consent. We do not screen vitamin D levels at baseline as part of the ToV5 eligibility criteria due to evidence from our observational cohort study of HIV-infected Tanzanian pregnant women which indicated there was no plateau in the beneficial effects of increased 25(OH)D up to 70 ng/mL [17].

### Vitamin D_3_ and placebo regimen

In the trial, pregnant women are randomized to receive of one of two regimens: 1) vitamin D_3_ (cholecalciferol) oral supplements containing 3000 IU taken daily from randomization at 12–27 weeks gestation until 12 months postpartum; or 2) a matching placebo regimen taken daily from randomization until 12 months postpartum.

The 3000 IU vitamin D_3_ regimen was selected in order to sustain 25(OH)D levels > 32 ng/mL for nearly all pregnant women while having minimal risk of hypercalcemia. In a landmark safety and effectiveness trial of vitamin D_3_ supplementation conducted by Hollis and Wagner, pregnant US women were randomized receive 400, 2000, or 4000 IU vitamin D_3_/day from 12–16 weeks gestation until delivery [30]. In this trial the proportion of women reaching a 25(OH)D concentration greater than 32 ng/mL at delivery was comparable for women in the 4000 IU (84%) and the 2000 IU (80%) arms but was significantly lower in the 400 IU (52%) arm. There were no differences in any safety measure between arms and the Data and Safety Monitoring Board (DSMB) determined no hypercalcemia events were attributed to vitamin D supplementation or circulating 25(OH)D levels. Based on these and other data, the US Institute of Medicine (IOM) set the tolerable upper intake limit (UL) of vitamin D_3_ to 4000 IU/day during pregnancy and lactation [31]. In ToV5 we decided to utilize 3000 IU vitamin D_3_ per day, as the studies the IOM used to set the 4000 IU tolerable upper limit were not conducted among pregnant women residing equatorially. We also restricted ToV5 enrollment to 12–27 weeks gestation due to a lack of safety data on the use of 3000 IU daily supplement units before 12 weeks of gestation. Vitamin D_3_ supplements (cholecalciferol) were chosen over vitamin D_2_ (ergocalciferol) supplements since vitamin D_3_ is more effective in increasing and sustaining circulating 25(OH)D [32].

The vitamin D_3_ and placebo regimen for ToV5 are produced in the USA by Tischcon Corp. (Salisbury, MD) and shipped to Tanzania. There is no discernible difference between vitamin D_3_ and placebo supplements in any manner, including appearance or taste. The placebo inactive ingredients included maltrin, gelatin, magnesium stearate, and titanium dioxide. The vitamin D_3_ and placebo supplements are hard gelatin capsules containing white powder which weigh on average 467 mg. Both regimens are also stored in identical bottles containing 45 capsules which are prelabeled with the patient ID. The vitamin D_3_ and placebo regimens have a shelf-life of 2 years, and thus two batches are required to complete the trial. In order to monitor vitamin D_3_ composition of the supplements throughout their shelf-life, Tishcon Corp. will test a random sample of capsules by high-performance liquid chromatography (HPLC) at production and then again at the end of the shelf-life after return shipping to the USA from Tanzania.

### Sample size

Sample size and power calculations for ToV5 were based on a two-sided test of proportions and a *z*-statistic using the asymptotic variances of the observed proportions which assumed 1:1 randomization to vitamin D_3_ and placebo arms and a nominal Type I error rate (alpha) of 0.05 [33]. We also assumed a 90% retention rate of HIV-infected mothers until study discharge at 1 year postpartum, 90% retention of birth outcomes (including fetal loss), and 85% retention of infants to 1 year postpartum (including fetal loss). We selected a trial sample size of 2300 individuals in order to provide adequate power for a reasonable range of incidences and effect sizes for the primary outcomes of maternal HIV progression and death, SGA births, and infant stunting. Table 1 presents the statistical power, varying the effect size and incidence of the primary outcomes. We will have > 80% power to detect an effect size of 0.70 or greater if the incidence of HIV progression or death is 15% over follow-up as expected. As for the SGA outcome (below tenth percentile birthweight for gestational age by sex on Oken standard [29]), a meta-analysis of published maternal vitamin D_3_ supplementation trials indicates a potentially strong effect on SGA with a relative risk of 0.67 (95% confidence interval (CI) 0.40–1.11) [34]. We will have > 80% power to detect a relative risk on SGA as low as 0.75, even if the risk of SGA is far lower than expected and equal to the reference population at 10%. As for infant stunting, in a recent RCT of multivitamins conducted among Tanzanian HEU infants, the risk of stunting at 1 year postpartum was about 20% [35]. As for the potential effect size of maternal vitamin D_3_ supplementation on infant stunting, the only randomized trial to date to examine linear growth was conducted in Bangladesh and found a large effect size on child stunting (odds ratio for stunting during first year 0.45, 95% CI 0.20–1.00) [36]. In ToV5 we will have adequate statistical power for infant stunting even if both the incidence of stunting and the effect size are unexpectedly smaller at 15% and a relative risk of 0.80, respectively.

**Table 1:** Statistical power for a trial sample size of 2300 with 1:1 randomization to vitamin D_3_ and placebo arms, and a nominal Type I error rate (alpha) of 0.05

Hazard ratio (HR) for maternal HIV progression (any increase in WHO HIV stage) or death			
Cumulative incidence over average 17 months follow-up^a^	HR = 0.60	HR = 0.65	HR = 0.70
12.5%	95.7%	88.3%	75.5%
15%	98.0%	93.0%	82.3%
17.5%	99.1%	95.8%	87.3%
Relative risk (RR) of small-for gestational age (SGA) birth			
Cumulative incidence of SGA in placebo arm	RR = 0.65	RR = 0.70	RR = 0.75
10%	98.4%	93.4%	81.2%
12%	99.5%	96.9%	88.3%
14%	99.9%	98.7%	93.0%
Relative risk (RR) of child stunting at 12 months of age (LAZ < –2)			
Cumulative incidence of stunting in placebo arm	RR = 0.60	RR = 0.70	RR = 0.80
15%	>99.9%	98.8%	78.4%
20%	>99.9%	>99.9%	90.3%
25%	>99.9%	>99.9%	96.3%

^a^Assumes the monthly incidence of maternal human immunodeficiency virus (HIV) progression or death is constant during follow-up

*LAZ* length-for-age *z*-score, *WHO* World Health Organization

### Enrollment and follow-up procedures

#### Screening and randomization procedures

The ToV5 has a two-part screening protocol which is integrated with routine PMTCT visits of the Dar es Salaam HIV care and treatment program. The trial is conducted at five PMTCT sites in Dar es Salaam, including Mnazi Mmoja Hospital, Buguruni Health Center, Mbagala Rangi Tatu Hospital, Sinza Hospital, and Tabata A Dispensary. These sites were selected since they are among the largest PMTCTs in Dar es Salaam in terms of patient numbers according to program records. All PMTCT sites are attached to ANC clinics. Research nurses and clinic staff were uniformly trained before the start of the study and have refresher meetings at least every 6 months or as issues arise.

At the first screening visit, research nurses identify HIV-infected pregnant women and assess eligibility criteria. Study nurses confirm pregnancy with a urine pregnancy test and gestational age is assessed through the report of the last menstrual period. Pregnant women receive HIV testing as part of routine antenatal care in Tanzania and women with unknown HIV status receive HIV testing with two licensed rapid assays [37]. If the HIV rapid assays are inconclusive (first test positive and second negative), the first and second HIV rapid assay are repeated following the same algorithm. If the results are still inconclusive upon repeat testing, the pregnant woman is advised that she may be in the acute HIV infection period and be asked to return for a repeat HIV testing in 2–4 weeks. Potential participants self-report the location of their residence and any plans to move out of the study area in the next year. Pregnant women who meet all other eligibility for ToV5 screening are then asked to provide written informed consent for a blood draw to screen for prevalent hypercalcemia. Serum calcium and albumin concentrations are assessed with the Roche Cobas Integra 400 Plus. Albumin-adjusted serum calcium concentration is then calculated using the following equation: calcium (mg/dL) = total calcium (mg/dL) + 0.8 × [4 − albumin (g/dL)] [38]. The results of the albumin-adjusted calcium screening are returned at a second screening clinic visit 1–2 weeks after the initial visit and any participants with above the normal physiologic range of albumin-adjusted calcium (>2.6 mmol/L) are diagnosed with hypercalcemia and are not eligible for ToV5 enrollment. For pregnant women with normal albumin-adjusted calcium concentrations, study nurses reconfirm enrollment eligibility, including being within the range of 12–27 weeks gestation, and written informed consent for trial enrollment is sought.

Pregnant women who consent for ToV5 enrollment are individually randomized 1:1 to the vitamin D_3_ or placebo arm. Randomization procedures were designed for complete allocation concealment and blinding of all participants, clinic staff, senior research staff, and trial statisticians. Trial arm allocation is performed according to a computer-generated randomization list (generated off-site by a nonstudy statistician) of 2300 individuals with sequence blocks of 10, stratified by study clinic. Two nonstudy statisticians will hold the randomization list codes until completion of primary analyses or until requested by the DSMB. The ToV5 DSMB reviews event rates every 6 months and will recommend any necessary changes to the trial sample size to maintain statistical power. All serious adverse events (SAEs) that are at least possibly related to the study regimen or activities are reported to all Institutional Review Boards within 24 h of Principal Investigator notification as per regulatory guidelines. An independent study pharmacist prepares the vitamin D_3_ and placebo regimen by labeling bottles with participant IDs based on the randomization list. At the randomization visit, allocation concealment is performed by assigning eligible pregnant women who consented for trial randomization to the next available numeric participant ID which corresponds to a set of prelabeled regimen bottles with the participant ID. As a result of these randomization procedures, all trial participants, research staff, and trial statisticians are not able to determine the allocated trial arm for any trial participant or identify trial participants who are on the same trial regimen. Allocation is completely concealed.

At the randomization visit, all study participants receive a full clinical examination from study physicians, they have their HIV disease stage assessed according to the WHO criteria, and all comorbidities are treated as per Tanzanian standard of care [39]. Nurses also administer a standardized questionnaire to collect information on sociodemographics, symptoms during the last 30 days (fever, cough, etc.), depression, anxiety, social support, and physical activity. The Hopkins Symptom Checklist (HSCL-25) will be administered to assess symptoms of depression and anxiety [40]. We have previously validated the HSCL-25 among Tanzanian women living with HIV against a diagnosis of a major depressive disorder by a trained psychiatrist using the Structured Clinical Interview for the Diagnostic and Statistical Manual of Mental Disorders, Fourth Edition (DSM-IV) [41]. We will measure functional dimensions of social support, a scale based on the Duke University–University of North Carolina Functional Social Support Questionnaire [42]. Nurses also collect anthropometric measurements including height, weight, mid-upper arm circumference (MUAC), and fundal height. All pregnant women also have their blood pressure assessed. Participants are also asked for cell phone numbers and the location of their home for follow-up visits, given the participant provides written informed consent. Home visits are conducted by trained research assistants who receive updated training on home visits in the context of HIV every 6 months. The accuracy of home visit information is assessed through spot checks by clinic supervisors. At the end of the randomization visit, participants are given a regimen bottle containing 45 capsules and are told to bring the bottle and remaining capsules to their next study visit.

#### Prenatal follow-up visits

All enrolled pregnant woman are seen in the research clinic every 4 weeks during pregnancy until delivery. Starting at 32 weeks gestation, all women also receive weekly phone calls and are instructed to call study staff when they go into labor. At all prenatal visits, study physicians perform a full clinical examination, assess WHO HIV disease stage, and treat all comorbidities in accordance with the Tanzanian standard of care. Nurses collect weight, MUAC, fundal height, and blood pressure. Study nurses also assess compliance with the trial regimen by counting the number of unconsumed capsules in returned regimen bottles. At the end of each visit participants are given a new regimen bottle containing 45 capsules of their randomized regimen. All women are given two regimen bottles containing 45 trial supplements starting at the 32-week gestation study visit to ensure an adequate supply for the period of delivery to the first study visit at 6 weeks postpartum. Pregnant women have a blood sample collected at 32 weeks gestation for assessment of albumin-adjusted calcium. Pregnant women with above the normal physiologic range of albumin-adjusted calcium (>2.6 mmol/L) are diagnosed with hypercalcemia and immediately stop the trial regimen for the duration of the study, and receive appropriate clinical management.

#### Labor and delivery assessment

Study nurses/midwives are available throughout the day and night to attend labor and delivery of participants. Deliveries which occur outside of Dar es Salaam are reached by phone to obtain relevant information from the mother and/or clinic staff. During this visit women and clinic staff are asked about any pregnancy complications since the last study visit and the duration of each stage of labor and complications of labor are recorded. Immediately after delivery, research midwives determine Apgar scores at 1 and 5 min. Length, weight, head circumference, chest circumference, and MUAC of the infant are also taken in triplicate. Infant weight is measured to the nearest 5 g using a digital scale (SECA, Hamburg, Germany) and length with the use of a rigid length board with an adjustable foot piece to 1-mm precision (SECA, Hamburg, Germany). Umbilical cord blood samples and a heel prick dried blood spot are collected from each infant.

#### Postnatal follow-up visits

Postnatal follow-up visits are integrated into standard PMTCT visits. Mother/child pairs visit the clinic at 6, 10, and 14 weeks postpartum and every month thereafter until trial discharge at 1 year postpartum. At the postnatal visits mothers receive a full clinical examination, have their WHO HIV disease stage assessed, and all comorbidities are treated in accordance with the Tanzanian standard of care. Nurses also collect weight, MUAC, and blood pressure from mothers. At the 6-month and 12-month postpartum visit the baseline questionnaire on sociodemographic, depression, anxiety, social support, and physical activity is readministered to mothers. All mothers have blood samples collected at 6 weeks, 6 months, and 12 months postpartum for assessment of albumin-adjusted calcium. Any mother with above the normal physiologic range of albumin-adjusted calcium (>2.6 mmol/L) is diagnosed with hypercalcemia and immediately stops the trial regimen for the duration of the study, and receives appropriate clinical management.

At postnatal visits infants receive a full clinical examination, have infant feeding practices assessed from the maternal report, and have morbidity history recorded with the aid of a pictorial morbidity diary that mothers are instructed to complete each day. The pictorial diary is used to document the occurrence of diarrhea, common cold, cough, difficulty breathing, fever, refusal to eat, drink, or breastfeed, pus draining from ears, and vomiting. At all study visits nurses also collect vaccination status, infant vital signs (temperature, respiratory rate, detection of chest indrawing, and blood pressure), and record any outpatient clinic visits or hospitalizations since the previous study visit. Study nurses also assess postpartum infant weight with a digital infant balance scale to the nearest 5 g (SECA, Hamburg, Germany) and length to 1-mm precision with a rigid length board with an adjustable foot piece (SECA, Hamburg, Germany). All infant anthropometric measurements are recorded in triplicate. To assess HIV status, all infants receive an HIV DNA PCR test at 6 weeks of age and then at 12 months postpartum. All infants have serum samples and dried blood spots stored for immunologic testing at 6 weeks, 6 months, and 1 year postpartum. At the trial discharge visit, child cognitive, motor, language, and socioemotional development will be assessed with the Caregiver-Reported Early Development Index (CREDI) [43] .

#### Laboratory investigations

In order to examine the effect of the vitamin D_3_ regimen on bone health, we will measure PTH in a 10% random sample of women at randomization, 32 weeks gestation, and then 6 weeks, 6 months, and 12 months postpartum. A 10% sample of children will also have PTH assessed at 6 weeks, 6 months, and 12 months postpartum. We will also assess the effect of vitamin D_3_ on maternal immune responses through measurement of IL-2, IL-12, IFN-γ, and cathelicidin in a random 10% sample of women at randomization, 32 weeks gestation, and then 6 weeks, 6 months, and 12 months postpartum.

#### Adherence assessment

In order to promote adherence to the trial regimen, all participants are encouraged to take the trial supplements at the same time every day, to put the bottle in a visible place, and identify an adherence assistant who would remind her to take the supplement. Compliance with the daily ToV5 supplements will be assessed in three ways ways: 1) direct questioning about use of the supplements in the previous 24 h and 2 weeks; 2) pill count from returned daily regimen bottles; and 3) biochemical assessment of 25(OH)D in a 10% subset of women longitudinally at randomization, 32 weeks gestation, and then 6 weeks, 6 months, and 12 months postpartum.

#### Standard of care

All study women and children are provided with HIV care and treatment that adhere to Tanzanian national PMTCT guidelines. The first-line ART regimen for HIV-infected pregnant women is tenofovir (TDF) + lamivudine (3TC) + efavirenz (EFV), and alternative first-line regimens include zidovudine (ZDV) + 3TC + EFV or ZDV + 3TC + nevirapine (NVP) if not well tolerated. Women receive cotrimoxazole prophylaxis if their CD4 T-cell count is < 200 cells/μL. Mothers receive daily iron (60 mg elemental) and folate (400 μg) supplementation in pregnancy and two doses of sulphadoxine pyremethamine (SP) are provided for malaria prophylaxis at 20–24 weeks and 28–32 weeks gestation. All infants will receive daily NVP from birth through 6 weeks postpartum irrespective of the infant feeding method. Cotrimoxazole prophylaxis is provided to all infants beginning at 4–6 weeks postpartum and continued until HIV infection is excluded (6 weeks after cessation of breastfeeding). Infants are tested for HIV infection at 6 weeks of age by PCR and, if negative, are retested again with PCR at 12 months. HIV-infected infants initiate ART regardless of WHO clinical stage or CD4 T-cell count. The current first-line ART regimen for HIV-infected children less than 3 years old in Tanzania is abacavir (ABC) + 3TC + lopinavir boosted by ritonavir (LPV/r). All infants receive immunizations and high-dose vitamin A supplementation (100,000 IU at 9 months) as per standard of care.

### Statistical analysis plan

An intent-to-treat analysis will be used as the primary analytic strategy for all analyses. The primary efficacy trial endpoints for the trial are: 1) maternal HIV progression or death from any cause; 2) SGA births; and 3) infant stunting at 12 months of age. The primary safety endpoint is maternal hypercalcemia. Maternal HIV progression will be defined as *any* increase in WHO HIV disease stage from the WHO HIV stage at randomization [39]. SGA will be defined as a birth weight less than the tenth percentile for gestational age by sex utilizing the Oken standard [29]. We acknowledge that the use of the last menstrual period (LMP) for gestational age dating may lead to misclassification of SGA [44]; nevertheless, errors in the maternal report of LMP are expected to be nondifferential with respect to the randomized regimen and therefore would lead to underestimation of the effect of vitamin D supplements on SGA. Stunting will be defined as LAZ which is 2 or more standard deviations below the WHO child growth standard reference median [45]. Hypercalcemia will be defined as an albumin-adjusted calcium > 2.6 mmol/L. We will use the log-rank test stratified by study clinic to assess differences in the incidence of maternal HIV progression or death and use the binomial proportion test for SGA births and child stunting at 12 months of age between the treatment arms. The Fisher’s exact test will be used to assess differences in the proportion of mothers with incident hypercalcemia.

Secondary endpoints for the trial include: 1) postrandomization maternal CD4 T-cell count and post-randomization HIV viral load; 2) postrandomization maternal immunologic biomarker levels (IL-2, IL-12, IFN-γ, and cathelicidin); 3) postrandomization maternal weight during pregnancy; 4) maternal depression and anxiety as assessed by the HSCL; 5) maternal hypercalcemia (serum albumin adjusted calcium > 2.6 mmol/L); 6) miscarriage; 7) stillbirth; 8) preterm birth; 9) birthweight; 10) low birthweight (<2500 g); 11) mother-to-child transmission of HIV; 12) infant mortality; 13) infant growth trajectory in the first year of life (LAZ, WAZ, and WLZ); 14) infant wasting (WLZ < –2); 15) infant underweight (WAZ < –2); 16) infant morbidities during the first year of life; 17) postrandomization infant cognitive, motor, and socioemotional development scores on the CREDI; 18) serum 25(OH)D levels in the mother and in the infant during the first 12 months of life; and 19) postrandomization maternal and infant PTH concentration. The binomial proportion test will be used for nonrepeatable binomial secondary outcomes while generalized linear mixed models with random intercepts, compound symmetric covariance structures, and robust standard errors will be used for repeated binomial secondary outcomes. Linear mixed-effects models with a random intercept, a compound symmetric covariance structure, and robust standard errors will be used to assess the effect of the vitamin D_3_ supplementation on the difference between baseline and postrandomization of continuous secondary endpoints.

We will examine effect modification of any treatment effect by predefined baseline variables: maternal age, maternal body mass index, socioeconomic status, gestational age at randomization, CD4 T-cell count, hemoglobin concentration, WHO HIV disease stage, ART regimen, duration of ART, duration of exclusive and any breastfeeding, and trial regimen adherence. To assess the statistical significance of each interaction, we will use the likelihood ratio test for risk-ratio homogeneity for primary and secondary nonrepeatable binomial outcomes and the score test in the linear mixed-effects and generalized linear mixed models for repeatable binomial outcomes and continuous longitudinal secondary outcomes.

## Discussion

Antiretroviral therapy coverage for HIV-infected pregnant women in sub-Saharan Africa continues to increase and is accelerating due to implementation and planned roll-out of Option B+ in the majority of high HIV burden countries [2]. Provision of lifelong ART for all HIV-infected pregnant women may nearly eliminate mother-to-child HIV transmission, but will also lead to exposure of about 1.5 million HEU children every year to triple antiretrovirals in utero [5]. HIV-exposed uninfected (HEU) infants are at increased risk of mortality, and exposure to triple antiretrovirals in utero may further increase the risk of small-for-gestational age births and linear growth faltering [6–8, 12]. Accordingly, the ToV5 was designed to examine vitamin D_3_ supplementation as a low-cost ART adjunct intervention which may improve maternal health, birth outcomes, and linear growth for HIV-infected Tanzanian pregnant women and their children.

A search of clinicaltrials.gov determined the only other clinical trial of vitamin D_3_ among HIV-infected individuals with mortality, HIV progression, or morbidity as primary or secondary outcomes is an ongoing study called the “Trial of Vitamin D in HIV Progression” (NCT01798680). This trial is also conducted by our research group and is a randomized, double-blind, placebo-controlled trial of vitamin D_3_ supplementation among HIV-infected men and nonpregnant women conducted in Dar es Salaam, Tanzania. As a result, the ToV5 will likely be the first randomized trial to determine the effect of vitamin D_3_ supplementation on mortality and HIV disease progression among HIV-infected pregnant women. An additional search determined at least two trials of maternal vitamin D supplementation trials for improvement of birth outcomes and child linear growth that are ongoing or completed and to be published. A randomized trial of a maternal (4000 IU daily) and neonatal (400 IU daily) vitamin D_3_ supplementation versus placebo on maternal and neonatal complications was completed in rural Pakistan and results are to be published (NCT01229189). In addition a randomized, double blind, placebo-controlled trial called the Maternal Vitamin D for Infant Growth (MDIG) study is currently being conducted in Dhaka, Bangladesh (NCT01924013) [46]. The primary aims of the MDIG are to determine the effect of a range of maternal vitamin D_3_ supplementation regimens in pregnancy (4200 IU/week, 16,800 IU/week, and 28,000 IU/week) on infant length at 1 year as compared with placebo and to determine the effect of a postpartum vitamin D_3_ regimen of 28,000 IU/week versus placebo on length at 1 year of age among infants of mothers who received 28,000 IU/week vitamin D_3_ during pregnancy. As a result, we will likely be the first trial to examine the effect of vitamin D supplementation on birth outcomes and linear growth in the African context and for infants born to HIV-infected mothers.

Overall, the ToV5 will provide causal evidence regarding whether vitamin D_3_ should be considered as an ART adjunct intervention to improve maternal health, birth outcomes, and linear growth for Tanzanian HIV-infected pregnant women and their infants. The trial will also provide evidence for the effect of vitamin D_3_ supplementation on a wide range of secondary maternal and child health outcomes. The trial results will be communicated in academic journals and at the national and local levels in Tanzania through policy briefs and dissemination meetings. The results of ToV5 will likely be generalizable to HIV-infected pregnant women and their children in similar resource-limited settings utilizing the Option B+ approach.

### Trial status

Trial enrollment started on 15 June 2015 and recruitment is ongoing as of 30 July 2017.

## Additional file

Additional file 1: Standard Protocol Items: Recommendations for Interventional Trials (SPIRIT) 2013 checklist. (PDF 131 kb)

## Abbreviations

25(OH)D: 25-Hydroxyvitamin D
3TC: Lamivudine
ABC: Abacavir
ART: Antiretroviral therapy
CREDI: Caregiver-Reported Early Development Index
DSMB: Data and Safety Monitoring Board
EFV: Efavirenz
HEU: HIV-exposed uninfected
HIV: Human immunodeficiency virus
HPLC: High-performance liquid chromatography
HSCL: Hopkins Symtpom Checklist
IFN: Interferon
IL: Interleukin
LAZ: Length-for-age *z*-score
LMP: Last menstrual period
LPV/r: Lopinavir boosted by ritonavir
MDH: Management and Development for Health
MDIG: Maternal Vitamin D for Infant Growth
MUAC: Mid-upper arm circumference
MUHAS: Muhimbili University of Health and Allied Sciences
NVP: Nevirapine
PMTCT: Prevention of mother-to-child transmission
PTH: Parathyroid hormone
RCT: Randomized controlled trial
SGA: Small-for-gestational age
TB: Tuberculosis
TDF: Tenofovir
ToV5: Trial of Vitamins-5
WAZ: Weight-for-age *z*-score
WHO: World Health Organization
WLZ: Weight-for-length *z*-score
ZDV: Zidovudine
